# Molecular and Clinical Repercussions of GABA Transporter 1 Variants Gone Amiss: Links to Epilepsy and Developmental Spectrum Disorders

**DOI:** 10.3389/fmolb.2022.834498

**Published:** 2022-03-02

**Authors:** Florian P. Fischer, Ameya S. Kasture, Thomas Hummel, Sonja Sucic

**Affiliations:** University of Calabria, Italy; University of Basilicata, Italy; University of Genoa, Italy; 1 Institute of Pharmacology, Medical University of Vienna, Vienna, Austria; 2 Department of Epileptology and Neurology, University of Aachen, Aachen, Germany; 3 Department of Neuroscience and Developmental Biology, University of Vienna, Vienna, Austria

**Keywords:** autism, Drosophila melanogaster, epilepsy, gamma-aminobutyric acid (GABA), GABA transporter 1, intellectual disability, protein folding, transporter disease variants

## Abstract

The human γ-aminobutyric acid (GABA) transporter 1 (hGAT-1) is the first member of the solute carrier 6 (SLC6) protein superfamily. GAT-1 (*SLC6A1*) is one of the main GABA transporters in the central nervous system. Its principal physiological role is retrieving GABA from the synapse into neurons and astrocytes, thus swiftly terminating neurotransmission. GABA is a key inhibitory neurotransmitter and shifts in GABAergic signaling can lead to pathological conditions, from anxiety and epileptic seizures to schizophrenia. Point mutations in the *SLC6A1* gene frequently give rise to epilepsy, intellectual disability or autism spectrum disorders in the afflicted individuals. The mechanistic routes underlying these are still fairly unclear. Some loss-of-function variants impair the folding and intracellular trafficking of the protein (thus retaining the transporter in the endoplasmic reticulum compartment), whereas others, despite managing to reach their bona fide site of action at the cell surface, nonetheless abolish GABA transport activity (plausibly owing to structural/conformational defects). Whatever the molecular culprit(s), the physiological aftermath transpires into the absence of functional transporters, which in turn perturbs GABAergic actions. Dozens of mutations in the kin SLC6 family members are known to exhort protein misfolding. Such events typically elicit severe ailments in people, e.g., infantile parkinsonism-dystonia or X-linked intellectual disability, in the case of dopamine and creatine transporters, respectively. Flaws in protein folding can be rectified by small molecules known as pharmacological and/or chemical chaperones. The search for such apt remedies calls for a systematic investigation and categorization of the numerous disease-linked variants, by biochemical and pharmacological means *in vitro* (in cell lines and primary neuronal cultures) and *in vivo* (in animal models). We here give special emphasis to the utilization of the fruit fly *Drosophila melanogaster* as a versatile model in GAT-1-related studies. Jointly, these approaches can portray indispensable insights into the molecular factors underlying epilepsy, and ultimately pave the way for contriving efficacious therapeutic options for patients harboring pathogenic mutations in hGAT-1.

## On The Rudiments of Gaba and Gats

The γ-aminobutyric acid (GABA) is a non-proteinogenic amino acid, first detected in the brain tissue in the 1950s (Awapara et al., 1950; Roberts and Frankel, 1950). It is known to play diverse physiological roles as a metabolite, neurotransmitter and neurotrophin (Waagepetersen et al., 1999). GABA is the principal mammalian inhibitory neurotransmitter, essential for counterbalancing neuronal excitability. Alterations in GABAergic signaling have been implicated in seizure generation (Roth and Draguhn, 2012; Kang, 2017). The GABA transporter 1 (GAT-1), encoded by the *SLC6A1* gene, is one of the main GABA transporters in the brain. It is responsible for the reuptake of GABA from the synaptic cleft, constituting a core component of GABAergic signaling. Recent mutations discovered in the *SLC6A1* gene have been linked to a range of neurodevelopmental disorders, including diverse epilepsy syndromes, intellectual disability (ID) and autism spectrum disorders (Goodspeed et al., 2020). The precise molecular culprits underlying the pathophysiological *SLC6A1* mutations are as yet quite unknown. Recent experimental evidence suggests reduced or abolished GABA uptake function as a common feature underlying the disease mechanism (Mattison et al., 2018; Mermer et al., 2021). Additionally, some of the mutations likely trigger folding defects, leading to retention of GAT-1 proteins in the endoplasmic reticulum (ER) (Wang et al., 2020; Mermer et al., 2021). Diseases arising from foldingdeficient variants of other solute carrier (SLC) 6 transporters are not without precedent: e.g., misfolded variants of the dopamine transporter (DAT, *SLC6A3*) and the creatine transporter 1 (CRT-1, *SLC6A8*) cause infantile/juvenile parkinsonism-dystonia and the creatine transporter deficiency syndrome, respectively (Farr et al., 2020; Bhat et al., 2021). Insights gained from studies of these closely related transporters may better our understanding of the molecular pathophysiology behind *SLC6A1*-related disorders, and considerably accelerate the development of novel precision medicine treatments.

## The Subfamily of Gaba Transporter Proteins

The human genome encodes four isoforms of GATs, which are designated GAT-1 (*SLC6A1*), BGT-1 (betaine/GABA transporter 1, *SLC6A12*), GAT-2 (*SLC6A13*) and GAT-3 (*SLC6A11*). It should be noted that GATs in humans and rats share the same nomenclature, whereas the corresponding GATs in mice are named differently, i.e., GAT1, GAT2, GAT3 and GAT4, respectively (Schousboe et al., 2014). The physiological role of these high-affinity transport proteins is to regulate the extracellular levels of GABA during synaptic transmission and under basal conditions (Scimemi, 2014). The reported K_M_ values for the human isoforms GAT-1, BGT-1, GAT-2 and GAT-3 are 11, 18, 8.1, and 0.56 μM, respectively (Rowley et al., 2012). BGT-1 is also able to carry betaine, whereas GAT-2 and GAT-3 exhibit an additional capacity to transport taurine and ß-alanine (Rowley et al., 2012).

The tissue expression atlas of GATs revealed that the predominant isoforms in the brain are GAT-1 and GAT-3, while GAT-2 and BGT-1 are found primarily in the liver and kidney (Zhou and Danbolt, 2013). GAT-1 is mainly localized to presynaptic GABAergic neurons and to a minor degree to distal astrocytic processes (Rowley et al., 2012). It is highly expressed in the cerebellum, basal ganglia, olfactory bulb, retina and interpeduncular nucleus (Scimemi, 2014). In contrast to GAT-1, GAT-3 is thought to be exclusively located on astrocytes (Zhou and Danbolt, 2013). It shows robust expression in the olfactory bulb, brainstem, thalamus and hypothalamus but only modest expression in the caudate-putamen, hippocampus, cerebral cortex and cerebellum (Minelli et al., 1996). In the brain, GAT-2 is present in the leptomeninges and ependyma, and to a lesser degree on cortical neurons and astrocytes (Conti et al., 1999). BGT-1 has only been detected in the leptomeninges (Zhou et al., 2012), cerebral cortex and hippocampus (Zhu and Ong, 2004).

## The Human Gaba Transporter 1 in the Spotlight

The human GAT-1 isoform, encoded on chromosome 3 (3p25.3), is composed of 599 amino acid residues, organized into twelve putative transmembrane (TM) segments, with cytoplasmic amino- and carboxyl-termini (Bennett and Kanner, 1997; Hoglund et al., 2005). It is predominantly localized to presynaptic terminals and to distal astrocytic processes (Minelli et al., 1995). GAT-1 is also found in cell bodies and dendrites for a short time period during cortical development (Yan et al., 1997). At some synapses in the cerebellum and hippocampus, the average membrane density of GAT-1 was estimated to be about 800–1,300 /μm^2^. Approximately 60% of the transporter molecules were shown to reside at the cell surface, whereas the remaining 40% seem to be located in the cytoplasmic regions of the cell (Chiu et al., 2002).

The translocation process of GABA via GAT-1 is electrogenic and coupled to the inward transport of two Na^+^ ions and one Cl^−^ ion. Accordingly, the translocation of one neutral GABA molecule is predicted to lead to a net influx of one positive charge (Scimemi, 2014). The functional role of GAT-1 has been extensively studied in genetic mouse models (Jensen et al., 2003; Chiu et al., 2005; Cai et al., 2006; Liu et al., 2007a; Liu et al., 2007b; Xu et al., 2008; Cope et al., 2009). GAT-1 knockout (KO) mice show elevated ambient GABA levels, which cause an increase in GABA-mediated tonic conductance due to overstimulation of extrasynaptic GABA_A_-receptors. Moreover, GAT-1-deficient mice display a decreased quantal GABA release as well as a reduced presynaptic GABA_B_-receptor function. These findings imply that GAT-1 deficiency leads to an enhanced tonic and a reduced phasic inhibition (Jensen et al., 2003). GAT-1 KO mice also display some behavioral patterns (e.g. tremor, ataxia and nervousness) that phenocopy the clinical side effects of the GAT-1 inhibitor tiagabine (Chiu et al., 2005), which is used as an add-on therapy in the treatment of partial-onset seizures (LaRoche and Helmers, 2004). Besides tiagabine, several other selective GAT-1 inhibitors have been developed to date, e.g., Cl-966, SKF89976 A and NO-711, which are lipophilic derivatives of either nipecotic acid or guvacine. In addition, numerous drugs have been identified as non-selective GAT inhibitors. These drugs include ß-alanine, betaine, (S)-SNAP-5114, (R)-EF1502, THPO, exo-THPO and NNC 05–2090 (Kristensen et al., 2011).

GAT-1 is regulated *via* multiple mechanisms including second messengers and protein-protein-interactions. These forms of regulation are thought to modulate the function of GAT-1 either by redistributing the transporter or by altering the GABA translocation rates (Chen et al., 2004). Activation of protein kinase C is associated with a down-regulation of GAT-1. In contrast, tyrosine phosphorylation has been shown to increase the surface expression of GAT-1 due to reduced internalization rates (Quick et al., 2004). Moreover, GAT-1 is regulated by extracellular GABA levels, which typically boosts cell surface expression of the transporter. Conversely, inhibitors of GAT-1 have been shown to decrease surface levels of GAT-1 (Bernstein and Quick, 1999). GAT-1 is also known to undergo regulation by the SNARE protein syntaxin 1A, which binds to the transporter’s amino-terminal region. This interaction promotes both an increase in cell surface expression and a decrease in GAT-1 protein turnover rates (Deken et al., 2000). Several groups have showed that GAT-1 forms oligomeric structures (Schmid et al., 2001; Moss et al., 2009). Although each monomer is able to translocate GABA independently (Soragna et al., 2005), oligomerization is a prerequisite for concentrative export from the ER compartment and subsequent trafficking to GATs’ eponymous site of action at the plasma membrane (Scholze et al., 2002).

## The Clinical Spectrum of Human Gat-1 Disease Variants

**Figure 1:**
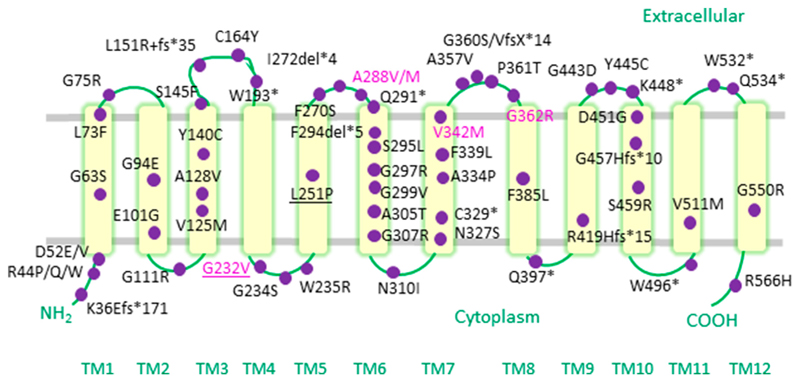
Epilepsy-associated variants mapped onto a human GAT-1 topology. Pathogenic point mutations in the *SLC6A1* gene reported in the literature to date are depicted as purple circles on a topology diagram of the human GAT-1. The mutations occur throughout hGAT-1, from transmembrane (TM) domains, to cytoplasmic amino- and carboxyl-termini, as well as intra- and extracellular loop regions. Recurrent mutations are indicated in the magenta font. Frameshift and termination codon mutations are indicated with “*fs*” and “*”, respectively. Pathogenic mutations found at equivalent conserved residues in other SLC6 transporters are underlined.

Over recent years, a compendium of *SLC6A1* mutations (Figure 1) have been associated with a range of neurodevelopmental disorders, including autism, variable degrees of ID and a spectrum of epilepsy syndromes (Table 1) (Carvill et al., 2015; Johannesen et al., 2018; Mattison et al., 2018; Goodspeed et al., 2020; Kahen et al., 2021). Point mutations in *SLC6A1* were first identified in patients suffering from epilepsy with myoclonic-atonic seizures (also known as Doose syndrome) (Carvill et al., 2015). This debilitating childhood-onset epilepsy syndrome is characterized by seizures of multiple types, such as myoclonic-atonic, atonic or generalized tonic-clonic seizures (Tang et al., 2020). Soon after, *SLC6A1* variants were also reported in individuals afflicted with other forms of generalized epilepsies (e.g., childhood absence epilepsy) as well as in some patients with focal epilepsies (e.g., temporal lobe epilepsy) (Johannesen et al., 2018). Detailed data on seizure semiology revealed that absence, atonic and myoclonic seizures are the most frequently observed seizure types (Johannesen et al., 2018; Goodspeed et al., 2020; Kahen et al., 2021).

Apart from epilepsy, mild to pronounced cognitive impairment is another common hallmark of *SLC6A1* variant carriers. In fact, almost all of the afflicted individuals display some degree of ID, mostly in the mild to moderate range (Johannesen et al., 2018; Goodspeed et al., 2020). A large fraction of the affected patients manifest behavioral problems, such as aggressive behavior/irritability, attention deficit, hyperactivity and autistic traits. Other reported clinical features include mild ataxia, unsteady gait, hypotonia, tremor and impairment of fine motor skills (Johannesen et al., 2018). Moreover, several mutations in *SLC6A1* have very recently been linked to a higher risk for autism and schizophrenia (Rees et al., 2020; Satterstrom et al., 2020). In the electroencephalogram, most patients exhibit generalized epileptiform discharges, especially at a frequency of 2–4 Hz. A generalized background slowing can be detected in one third of the cases (Goodspeed et al., 2020).

The currently available data guiding clinical management of *SLC6A1*-related disorders is rather scarce despite the large unmet need for effective treatment strategies of patients suffering from these conditions. Johannesen et al. reported that 20 of 31 patients achieved some seizure relief, with valproic acid being the most effective drug (Johannesen et al., 2018). However, seizure control was not correlated with the cognitive outcome, and on top of the broad spectrum of unpleasant adverse effects of valproic acid, make this compound a suboptimal therapeutic choice. Notably, significant improvements have been observed in response to a ketogenic diet (Carvill et al., 2015; Palmer et al., 2016), an avenue worth delving into.

The exact prevalence of *SLC6A1*-related disorders is difficult to estimate. However, it is important to note that epidemiological data reported for other solute carriers, e.g. the glucose transporter 1 (GLUT-1, *SLC2A1*), which is also linked to epilepsy and other neurological conditions, indicate a frequency of GLUT-1 mutations of approximately 1:83,000 in the Danish population (Larsen et al., 2015).

## Molecular Traits Behind SLC6A1 Variant Pathophysiology: The Rules and Lessons Drawn From the SLC6 Relatives

**Figure 2:**
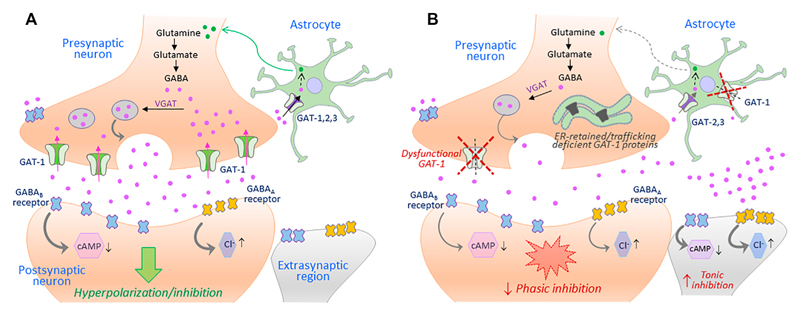
The putative repercussions of hGAT-1 variants at the GABAergic synapse. A simplified schematic showing the GABAergic synapse in a regular physiological state (**A**) and in a hGAT-1-triggered epilepsy setting (**B**). The GABAergic homeostasis is tightly regulated by the neuronal and glial GABA transporters. The absence of plasmalemmal hGAT-1 affects the extracellular clearance of GABA, which results in increased extrasynaptic GABA levels and reduced presynaptic GABA pools affecting the subsequent phasic neurotransmission. The higher levels of extrasynaptic GABA act on the extrasynaptic GABA_A_ and GABA_B_ receptors, inducing tonic inhibition.

Disease mutations can impair protein folding and trap transporter proteins in the ER compartment, thus precluding their export and intracellular trafficking. Other mutations emanate structural defects and disrupt transport activity without altering cell surface expression of the resulting proteins. Putative effects of such loss-of-function hGAT-1 variants, as currently understood, are depicted in Figure 2. To date, dozens of pathological transporter variants have been verified as folding-deficient. The first reported case of a misfolded SLC6 transporter was a variant of the human norepinephrine transporter (NET, *SLC6A2*). A 33-year-old woman suffering from the autonomic disorder orthostatic intolerance was found to harbor a heterozygous A457P point mutation in the *SLC6A2* gene (Shannon et al., 2000). The mutation compromised ER export, causing a substantial loss of cell surface expression. Moreover, it exerted a dominant-negative effect on the wild type transporter (i.e., product of the healthy allele) through formation of non-productive oligomeric complexes, targeted to degradative pathways (Hahn et al., 2003). This was consistent with the hypothesis that oligomer formation is a crucial requirement for ER export (Scholze et al., 2002). In DAT (*SLC6A3*), dozens of point mutations trigger infantile parkinsonism (Kurian et al., 2009; Ng et al., 2014). A vast majority of these induce DAT misfolding, i.e. the transporters accumulate as ER-resident core-glycosylated proteins (Mazhar Asjad et al., 2017). The genetic transmission is reported as autosomal recessive in all cases (i.e., patients are either homozygotes or compound heterozygotes), suggesting that clinical phenotypes only occur in the total absence of a functional DAT. Some variants exhibited a low residual uptake: e.g., A314V-DAT retained 8% of wild type DAT uptake levels. In contrast to mutants that were completely devoid of uptake activity, this variant led to a later disease onset and a milder clinical course. Hence, residual activity of the mutant transporters relates to the onset and the severity of the disease symptoms (Ng et al., 2014). In the instance of the glycine transporter 2 (GLYT-2, *SLC6A5*), several mutations have been linked to hyperekplexia/startle disease (Rees et al., 2006; Carta et al., 2012). Most mutations are transmitted in a recessive manner. However, some dominantly-inherited mutations have also been reported. At least one of the identified variants (S510R-GLYT-2) is known to accumulate in the form of intracellular aggregates, indicative of a folding defect (Rees et al., 2006). In addition, mutations in CRT-1 (*SLC6A8*) cause ID and epilepsy (Salomons et al., 2001; Van De Kamp et al., 2014). Confocal microscopy experiments revealed that many of these variants are trapped in the ER, i.e. co-localized with the ER marker calnexin (El-Kasaby et al., 2019).

Folding-deficient mutants can be rescued by chemical or pharmacological chaperones (Chaudhuri and Paul, 2006). These small molecules stabilize the misfolded protein, promote folding and facilitate delivery to the required cellular locations (Loo and Clarke, 2007). Chemical chaperones such as glycerol, dimethyl sulfoxide and 4-phenylbutyric acid (4-PBA) enhance folding of many proteins (Perlmutter, 2002). Pharmacological chaperones bind directly to, and stabilize, their cognate target proteins and their action is restricted to specific target proteins. Prominent examples include migalastat and lumacaftor, used to treat Fabry disease (Germain et al., 2016) and cystic fibrosis (Wainwright et al., 2015), respectively. The first pharmacochaperone shown to be highly effective in the SLC6 transporter family was (nor)ibogaine. We showed that ibogaine binds to the inward-facing transporter conformation and rescues the misfolded serotonin transporter (SERT, *SLC6A4*) mutant R607A/I608A-SERT, which harbors mutations in the ER-export motif (El-Kasaby et al., 2014; Montgomery et al., 2014). Introducing second site suppressor mutations, which trap SERT in the inward-facing state, also promoted surface expression of folding-deficient SERTs (Koban et al., 2015). Noribogaine and its congeners also rescued several misfolded parkinsonism-causing DAT variants (Beerepoot et al., 2016; Mazhar Asjad et al., 2017). Partial substrates like PAL1045 can rescue the starkly misfolded P601A/G602A-SERT (Bhat et al., 2017). Chemical chaperones such as 4-PBA rescued CRT-1 variants linked to ID (El-Kasaby et al., 2019). Moreover, heat shock protein (HSP) inhibitors proved efficient: the HSP70 inhibitor pifithrin-μ rescued misfolded DATs, while the HSP90 inhibitor 17-dimethylaminoethylamino-17-demethoxygeldanamycin (17-DMAG) sensitized misfolded SERTs to the pharmacochaperone action of noribogaine (Kasture et al., 2016; Mazhar Asjad et al., 2017). The action of HSP inhibitors can be rationalized: the folding trajectory is monitored by a relay of HSPs. These proteinaceous chaperones must be released for the protein cargo to exit the ER. Their inhibition relaxes the stringent ER quality control and promotes ER export (Freissmuth et al., 2017). Pharmacochaperoning is not limited to heterologous expression in cell lines; we also provided a proof-of-principle that folding-deficient DATs are amenable to rescue *in vivo*, in *Drosophila melanogaster* (Mazhar Asjad et al., 2017).

The above inferences may well echo onto the hGAT-1 epilepsy variants, considering the high phylogenetic similarity in the SLC6 family (Freissmuth et al., 2017; Bhat et al., 2021). As a matter of fact, it is striking that pathogenic mutations can occur at conserved/equivalent residues among members of the SLC6 transporter family. For instance, a substitution of alanine at position 275 in the human GLYT-2 to threonine (i.e. variant A275T-hGLYT-2, equivalent to A288-hGAT-1 shown in Figure 1) leads to hyperekplexia/startle disease. The molecular grounds for disease onset, discerned using electrophysiological measurements, revealed that A275T induces a reduction in Na^+^ion affinity and in turn diminishes the voltage-sensitive glycine uptake (Carta et al., 2012). A similar scenario transpires for the recurring hGAT-1 variant G232V. In the human CRT-1, the substitution of the corresponding glycine residue by an arginine (i.e. variant G253R-hCRT-1) triggers ID accompanied by severe delay in speech and language development. Reportedly, the affected boy’s carrier mother (i.e. creatine transporter deficiency being an X-linked disease) also exhibited borderline intellectual functioning (Battini et al., 2011). At the molecular level, we found that the G253R mutation elicits its loss-of-function disease phenotype by triggering protein folding defects in the hCRT-1 protein, trapping the mutated transporter in the ER (El-Kasaby et al., 2019). Although the cell surface expression of this variant was restored upon treatment with the chemical chaperone 4-PBA or inhibitors of the HSP 70 and 90 (pifithrin-μ and 17-DMAG, respectively), its creatine uptake activity was not salvaged to any appreciable level (El-Kasaby et al., 2019).

## Animal Models in Exploring SLC6A1 Disorders: an Emphasis on Fruit Flies

The GABA transporter is evolutionarily highly conserved. *SLC6A1* orthologs exist in organisms ranging from roundworms and fruit flies to zebrafish and mammals. Various animal models have been explored to understand the pathophysiological aspects of epilepsy (Engel and Wu, 1994; Avoli, 1995; Pavlidis and Tanouye, 1995; Lee and Wu, 2002; Noebels, 2003; Williams et al., 2004; Baraban et al., 2005). Reduced or altered GAT-1 functioning in mice results in absence seizures, and thalamic GAT-1, which exhibits marked astrocytic expression, is known to regulate absence seizures (Cope et al., 2009). A library of transgenic mice (expressing multiple GAT-1 variants) would be an ideal approach to study GAT-1 disease-associated pathological changes, as well as drug candidate screenings. However, establishing such libraries is not only laborious, but also logistically and financially challenging.

In contrast to their vertebrate counterparts, invertebrates, such as roundworms and fruit flies, possess only a single GABA transporter. As such, they provide a unique opportunity to study disease-relevant mutants in a high-throughput manner. We here focus on utilizing *Drosophila melanogaster* as a model organism to unravel the pathophysiological aspects of GAT-1 variants. These dew-loving fruit flies have remained an organism of choice in studies of conserved biological processes for over 100 years. This is largely on account of their short life cycle, ease of maintenance, cost-effectiveness and their rich genetic arsenal. Around 75% disease-related genes carry an ortholog in flies (Reiter et al., 2001). The ability to generate transgenic flies that express human proteins in a spatial and temporal manner, makes *Drosophila* ideal in examining human disorders (Rubin and Spradling, 1982; Brand and Perrimon, 1993; Gratz et al., 2015; Mazhar Asjad et al., 2017). *Drosophila* has gained much attention in studies of conserved solute carrier proteins (Thimgan et al., 2006; Kasture et al., 2016; 2017; 2018; 2019; Sucic et al., 2016). It recently proved to have great translational potential in the case of folding-impaired DAT variants (Mazhar Asjad et al., 2017). We, and others, have examined the trafficking and activity of dopamine transporter deficiency syndrome (DTDS)-linked mutants in *Drosophila* (Kasture et al., 2016; Mazhar Asjad et al., 2017; Aguilar et al., 2021). Drug screens carried out in *Drosophila* were led by data from in *silico* and *in vitro* experiments, and have also been validated in induced pluripotent stem cells (iPSCs) obtained from DTDS patients (Ng et al., 2021).

**Figure 3:**
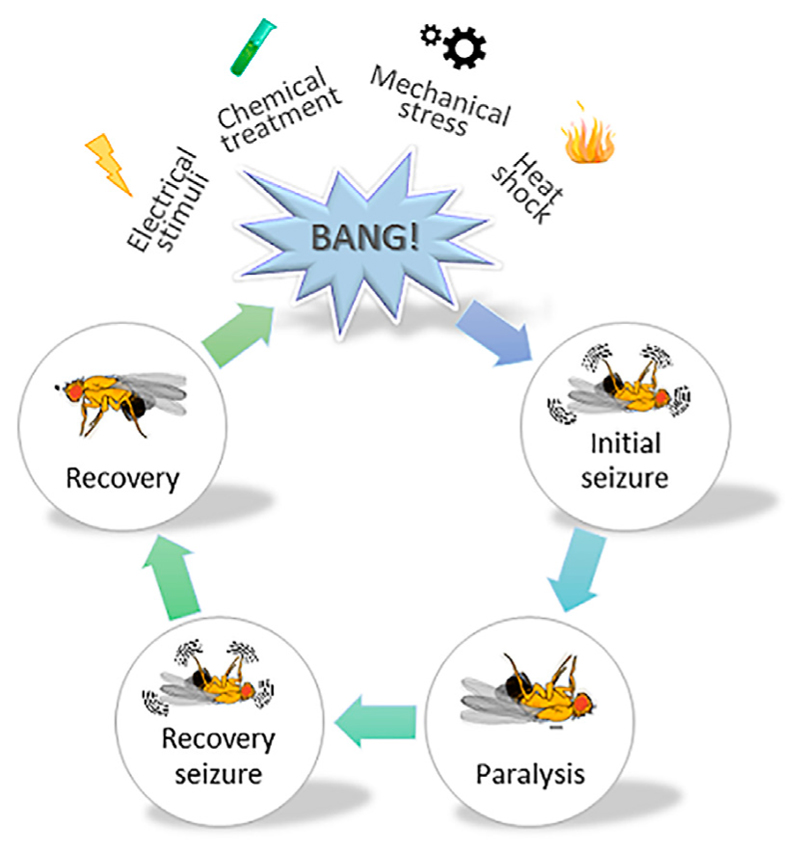
A schematic illustrating the use of fruit flies in studying epilepsy. A cartoon depicting stereotypical behavior observed in *Drosophila melanogaster*, subjected to mechanical stress. Various stressors such as mechanical, chemical, electrical and heat shock are typically used to induce seizure-like activity in fruit flies. They show varying degrees of sensibility to seizure and duration thereof, depending on the genetic susceptibility. Vortexing flies for a brief 10 s period induces initial seizure-like activity characterized by leg twitches, abdominal contractions, proboscis extensions and wing flapping. The initial phase is followed by the paralysis phase and a subsequent recovery phase, whereby the fly tries to regain its posture. The time required by the fly to fully regain its posture (i.e., enter the recovery phase) is collected for data analysis.

The *Drosophila* GAT (dGAT) is expressed exclusively on astrocytes (Stork et al., 2014). Surface dGAT expression is highly dynamic and regulated by metabotropic GABA receptor signaling (Muthukumar et al., 2014). The excitatory amino acid transporter, which takes up glutamate, is also exclusive to astrocytic expression in flies (Soustelle et al., 2002). The GAT-KO or null mutation in flies leads to embryonic lethality. However, this phenotype is rescuable *via* expression of dGAT in astrocytes (Stork et al., 2014). A knockdown of dGAT during the development induces severe locomotor defects in fruit flies, at both larval and adult stages (Stork et al., 2014). One study reported that impaired glutamate/GABA/glutamine cycling in adult *Drosophila* astrocytes results in motor defects and greatly increases the recovery time from heat-induced seizures, both of which can be appreciably rescued by overexpressing dGAT in astrocytes (Mazaud et al., 2019). In other words, GAT expression, when modulated only in the adult stage, can affect the locomotor activity and seizure sensibility in flies. Similar to mammals, where GABA_B_ agonists induce absence seizures and GABA_B_ inhibitors block them, a reduction of astrocytic metabotropic GABA_B_ signaling ameliorates the seizure activity in flies (Muthukumar et al., 2014). The *Drosophila* model is not a new player in the epilepsy field: the role of diverse ion channels in the generation of epilepsy were discovered using fruit flies (Kuebler and Tanouye, 2000 Ganetzky and Wu, 1982, reviewed in Ganetzky, 2000; Song and Tanouye, 2008). Henceforth, *Drosophila* has remained the model organism of choice when it comes to defining the molecular underpinnings behind generalized epilepsy (Ghosh et al., 2018; Manivannan et al., 2021; Yap et al., 2021). A simplified illustration on the use of fruit flies in epilepsy-related research is shown in Figure 3. Upon mechanical agitation, by brief 10-s vortexing, *Drosophila* exhibit stereotypical seizure-like activity characterized by leg twitches, abdominal contractions, proboscis extensions and wing flapping, which is followed by paralysis, delayed spasms (recovery seizures) and recovery to normal posture. Genetic background largely affects the sensibility to seizures and seizure-duration in flies, whereby bang-sensitive mutants exhibit longer recovery times. In addition to mechanical stimulus, seizure-like activity can also be induced by heat shock (i.e., exposure to high temperature), high-frequency electrical stimulation, and chemical treatment (i.e., picrotoxin feeding) (Ganetzky and Wu, 1982; Pavlidis and Tanouye, 1995; Stilwell et al., 2006).

Flies are deemed an attractive model for high-throughput screening of antiepileptic drugs (Stilwell et al., 2006). dGAT and hGAT-1 show 52% sequence similarity, and remarkably, most of the disease-relevant amino acid residues are conserved among the two proteins. Novel gene editing tools such as CRISPR/Cas9 technique can be employed to create disease point mutations in the dGAT sequence (Lamb et al., 2017). Humanized flies expressing GAT-1 pathogenic variants could also be easily generated, and their trafficking through the secretory pathway and functioning at the plasmalemma subsequently examined in neuronal and astrocytic populations in flies. It is crucial to understand the fate of misfolded GAT-1 variants in GABAergic neurons and astrocytes. The mechanisms of how reduced (or totally absent) surface expression of GAT-1 affects the functional tripartite synapse can be addressed by assessing changes in synaptic connectivity (Shearin et al., 2018) and activity in flies (Macpherson et al., 2015). Flies also allow for inspecting whether the ER-retained fraction of GAT-1 proteins undergoes rapid clearance or imparts ER-stress (Ryoo et al., 2013). Additionally, a GABA biosensor can be utilized to evaluate the changing trends of extracellular GABA levels (Marvin et al., 2019), whilst GAT-1 activity can be assessed in a sensitized background for locomotor functioning and susceptibility to seizures.

## Is the Gain-of-Function Brunt of the Gabaergic System to Blame?

Evidently, the molecular rationale underlying *SLC6A1*-related disorders is not entirely clear. GAT-1 KO mice provided several valuable insights: 1) they are more sensitive to pentylenetetrazole–induced seizures and display spontaneous spike-and-wave discharges (SWD), which are typically associated with absence seizures (Chiu et al., 2005; Cope et al., 2009). 2) They show an increased extrasynaptic GABA_A_ receptor-mediated tonic conductance in thalamic, cerebellar and cortical brain regions (Chiu et al., 2005; Bragina et al., 2008; Cope et al., 2009). In other words, the tonic inhibition imparted by peri- or extrasynaptic GABA_A_ receptors is altered in GAT-1 KO mice. 3) Aberrant phasic inhibition is observed in thalamic and cortical regions (Bragina et al., 2008; Cope et al., 2009). This observation is contrary to other absence seizures models, where phasic inhibition remained unchanged with the tonic inhibition only being affected (Cope et al., 2009).

The role of thalamic GAT-1 in modulating absence seizure was studied in wild type Wistar rats by intrathalamic administration of the selective GAT-1 inhibitor NO-711. This inhibition induced absence seizures and was rescued by ethosuximide, indicating that thalamic GAT-1 is crucial in modulating absence seizures (Cope et al., 2009). Abundantly available extracellular GABA acts on extrasynaptic GABA_A_ receptors in the thalamocortical region to induce absence seizure. The δ subunit of extracellular GABA_A_ receptors is linked to aberrant tonic inhibition, and gain-of-function mutations in the *GABRD* gene encoding the δ subunit, mimic the phenotypic spectrum of patients harboring *SLC6A1* disease mutations (AhriNg et al., 2021). Furthermore, GABA_B_ receptor agonists are known to induce absence seizures and can even facilitate the extrasynaptic GABA_A_ receptor-mediated tonic inhibition (Cope et al., 2009). The GABA_B_ receptor-mediated absence seizures are linked to the activation of low-voltage-activated (T-type) calcium channels in the thalamus (Kim et al., 2001). Whether T-type calcium ion channels are affected in *SLC6A1*-related disorders remains unclear. In a nutshell, the thalamus is a region critical to controlling absence seizures, with GAT-1 exclusively expressed on astrocytes, and reduced GAT-1 function and/or enhanced GABA_A_ and GABA_B_ receptor tonic activation precipitating in absence seizures.

The mutations in *SLC6A1* reduce or abolish GABA uptake and in a clinical setting they appear to phenocopy the GAT-1 KO mice behavioural defects. All known mutations linked to *SLC6A1*-related disorders exhibit variable degrees of ER retention, suggesting that the GAT-1-mediated uptake is partly or completely affected (Mermer et al., 2021). As a consequence, if the downstream signalling mediated by GABA_A_ and GABA_B_ receptors in phasic (synaptic) and/or tonic (extrasynaptic) manner is altered, calls for further investigation. The GAT reportedly maintains GABA homeostasis by uptake and release of the neurotransmitter (Wu et al., 2007). How exactly extracellular GABA levels are maintained and cleared in the absence of functional GAT-1, remains to be clarified. Folding-impaired variants might incur additional ER stress and so further exacerbate the convoluted pathophysiology of *SLC6A1* disorders, many aspects of which ought to be brought to light by imminent *in vitro* and *in vivo* models of the disease.

## Concluding Remarks

The transporter research community is faced with an escalating amount of reports linking pathological conditions in people with specific variants in transporter genes. We here aimed to convey the impending clinical impact of probing the molecular core of such disorders, ideally at the level of each individual mutation. The pharmacotherapeutic potential of such in-depth studies is immense: it can translate into shaping the long-awaited strategies for adequate treatment of severe diseases, such as epilepsy, ID or parkinsonism, to name just a few. A systematic and rational search for novel therapeutic options by pharmacological means, i.e., treatment with small molecules (e.g., chemical/pharmacological chaperones or allosteric modulators) to restore the activity of dysfunctional variants has proven worthwhile in the paradigm of DAT variants associated with DTDS. Very recently, gene therapy was employed to restore DAT expression and ameliorate pathophysiology in iPSC and mouse models of this condition (Ng et al., 2021). With respect to GAT-1-linked syndromes, the epileptogenic mechanisms are still not utterly clear-cut. While some mutations appear to impair protein folding and/or trafficking, others trigger structural/conformational defects, with both scenarios irrefutably ending in deficient GABA transport. State-of-the-art computer simulation models can serve as another expedient complimentary approach in exploring mutation-specific ramifications at the atomic level, backing the biochemical and pharmacological data. Seminal discoveries from studies on other SLC6 family members (e.g., SERT, DAT and CRT-1) provide ample succour in facing the challenges of GAT-1 disease variants, and grant an optimistic outlook on finding the cure. In view of our recent work, we begin to appreciate how small molecules can become auspicious therapeutic agents in tackling great medical obstacles.

## Figures and Tables

**Table 1:** Human GAT-1 variants associated with neurological disorders.

Variant	Associated Phenotype(s)	References
K36Efs*171 (*de novo*)	Early onset absence epilepsy, moderate ID, hypotonia	Johannesen *et al*. (2018)
R44Q (*de novo*)	Epilepsy with myoclonic-atonic seizures, mild ID, autistic features	Carvill *et al*. (2015)
R44W (*de novo*)	Epilepsy, autism spectrum disorder, hypotonia	Kahen *et al*. (2021), Mermer *et al*. (2021)
D52 E/V (*inherited/AD*)	Global developmental delay	Landrum *et al*. (2018), NCBI ClinVar ID 987287/987286
F53S (*inherited/AD*)	Global developmental delay	Landrum *et al*. (2018), NCBI ClinVar ID 987288
G63S (*de novo*)	ID, developmental disorder	Liu *et al*. (2018)
L73 F (*de novo*)	Epilepsy	Mermer *et al*. (2021)
G75R (*de novo*)	Generalized epilepsy, mild ID	Johannesen *et al*. (2018)
G94 E (*unknown*)	Epilepsy	Mattison *et al*. (2018)
E101G (*de novo*)	Epilepsy, language disorder, developmental delay, ID, autism spectrum disorder, hypotonia, movement disorder	Islam *et al*. (2018), Kahen *et al*. (2021)
G111R (*de novo*)	Language disorder, developmental delay, hypotonia, movement disorder	Kahen *et al*. (2021)
V125M (*gonadal mosaic*)	Epilepsy with myoclonic-atonic seizures, moderate ID, ADHD	Poliquin *et al*. (2021)
A128V	ID, developmental disorder	Liu *et al*. (2018)
Y140C (*de novo*)	Epilepsy with myoclonic-atonic seizures, mild to moderate ID	Johannesen *et al*. (2018)
S145 F (*de novo*)	Mild ID, autism spectrum disorder, irritability, mild hypotonia, ataxia, chorea	Johannesen *et al*. (2018)
L151R + fs*35 (*de novo*)	ID, myoclonic-atonic seizures	Rauch *et al*. (2012)
C164Y (*de novo*)	Epilepsy with myoclonic-atonic seizures	Palmer *et al*. (2016)
W193* (*de novo*)	Epilepsy with myoclonic-atonic seizures, mild ID, mild autistic traits	Carvill *et al*. (2015), Johannesen *et al*. (2018)
G232V (*maternal and de novo*)	Epilepsy with myoclonic-atonic seizures (evolving to atypical benign epilepsy with centrotemporal spikes in one patient), mild to moderate ID and learning disabilities, mild ataxia	Johannesen *et al*. (2018)
G234S (*unknown*)	Lennox-Gastaut syndrome, moderate ID	Cai *et al*. (2019) Mermer *et al*. (2021)
W235R (*unknown, adopted*)	Absence epilepsy, moderate ID, autism spectrum disorder	Mattison *et al*. (2018)
L251P (*de novo*)	Language disorder, developmental delay, ID, hypotonia	Kahen *et al*. (2021)
F270S (*de novo*)	Generalized epilepsy, mild ID, irritability, ADHD	Johannesen *et al*. (2018), Mattison *et al*. (2018)
I272del*4 (*de novo*)	Epilepsy with myoclonic-atonic seizures, moderate ID, bilateral upper extremity tremor, mild tandem gait, ataxia	Mattison *et al*. (2018)
A288M (*de novo*)	Lennox-Gastaut syndrome, developmental delay, ID, autism spectrum disorder	Cai *et al*. (2019)
A288V (*inherited and de novo*)	Epilepsy with myoclonic-atonic seizures, atypical benign epilepsy with centrotemporal spikes (evolving into a generalized epilepsy), mild to severe ID, autistic features, aggressive behavior	Sanders *et al*. (2012), Carvill *et al*. (2015), Johannesen *et al*. (2018)
Q291* (*de novo*)	Epilepsy, language disorder, developmental delay, hypotonia	Kahen *et al*. (2021)
F294del*5 (*de novo*)	Epilepsy with myoclonic-atonic seizures, moderate ID, attention deficit, mild ataxia	Johannesen *et al*. (2018)
S295L (*de novo*)	Epilepsy, developmental delay, movement disorder, hypotonia	Kahen *et al*. (2021), Mermer *et al*. (2021)
G297R (*de novo*)	Epilepsy with myoclonic-atonic seizures, severe ID, autistic features, moderately severe tremor, aggressive behavior	Carvill *et al*. (2015)
G299V (*de novo*)	Autism spectrum disorder	Wang *et al*. (2016), Mermer *et al*. (2021)
A305T (*unknown*)	Epilepsy, language disorder, developmental delay, hypotonia	Kahen *et al*. (2021), Mermer *et al*. (2021)
G307R (*de novo*)	Epilepsy, language disorder, developmental delay, hypotonia, Rett-like syndrome	Lucariello *et al*. (2016), Kahen *et al*. (2021)
N310I (*de novo*)	ID, developmental disorder	Liu *et al*. (2018)
N327S (*de novo*)	Epilepsy, language disorder, developmental delay, ID, autism spectrum disorder, hypotonia, movement disorder	Kahen *et al*. (2021)
C329* (*de novo*)	Epilepsy with myoclonic-atonic seizures, mild ID, aggressive behavior	Johannesen *et al*. (2018)
S331G (*de novo*)	Epilepsy, language disorder, developmental delay, ID, ADHD, hypotonia, movement disorder	Kahen *et al*. (2021)
A334P (*mosaic mother*)	Epilepsy with myoclonic-atonic seizures, moderate ID	Carvill *et al*. (2015)
F339L (*de novo*)	Autism spectrum disorder	Yuen *et al*. (2016)
V342M (*paternal and de novo*)	Childhood absence epilepsy, epilepsy with myoclonic-atonic seizures, eyelid myoclonia with absences, generalized epilepsy, mild to severe ID and learning disabilities, autism spectrum disorder, aggressive behavior, ADHD, tremor, mild hypotonia, weak fine motor skills, ataxia	Johannesen *et al*. (2018)
A357V (*de novo*)	Epilepsy with myoclonic-atonic seizures, moderate ID, unsteady gait	Johannesen *et al*. (2018)
G360S/VfsX*14 (*unknown*)	Autism spectrum disorder	Wang *et al*. (2016)
P361T (*de novo*)	Generalized epilepsy, autism spectrum disorder	Wang *et al*. (2020), Mermer *et al*. (2021)
G362R (*mosaic mother*)	Lennox-Gastaut syndrome, temporal lobe epilepsy, moderate ID	Halvorsen *et al*. (2016), Johannesen *et al*. (2018)
F385L (*de novo*)	Epilepsy with myoclonic-atonic seizures, mild to moderate ID, autism spectrum disorder	Johannesen *et al*. (2018)
Q397* (*de novo*)	Epilepsy, autism spectrum disorder	Wang *et al*. (2016)
L408Wfs*26 (*unknown*)	Epilepsy, developmental delay, ADHD, hypotonia	Kahen *et al*. (2021)
R419Afs*15 (*unknown*)	Epilepsy, developmental delay, ADHD, autism spectrum disorder, hypotonia, movement disorder	Kahen *et al*. (2021)
Y445C (*unknown*)	Generalized epilepsy	Mattison *et al*. (2018)
G443D (*de novo*)	Epilepsy, developmental delay, autism spectrum disorder	Devries *et al*. (2020)
K448* (*de novo*)	Epilepsy with myoclonic-atonic seizures, moderate ID (nonverbal), autism spectrum disorder, unsteady gait	Johannesen *et al*. (2018)
D451G (*de novo*)	Moderate ID, autism spectrum disorder, speech delay and seizures	Bowling *et al*. (2017)
G457Hfs*10 (*de novo*)	Epilepsy with myoclonic-atonic seizures (evolving to atypical benign epilepsy with centrotemporal spikes), mild ID, unsteady gait/balance problems	Carvill *et al*. (2015), Johannesen *et al*. (2018)
S459R (*de novo*)	Generalized epilepsy, severe ID (almost nonverbal), aggressive behavior	Johannesen *et al*. (2018)
W496* (*unknown*)	Generalized epilepsy, autism spectrum disorder, mild hypotonia	Mattison *et al*. (2018)
V511M (*de novo*)	Generalized epilepsy, mild ID (verbal), ADHD	Johannesen *et al*. (2018)
W532* (*unknown*)	Epilepsy, language disorder, developmental delay, autism spectrum disorder, hypotonia, movement disorder	Kahen *et al*. (2021)
Q534* (*de novo*)	Epilepsy with myoclonic-atonic seizures, mild ID, mild ataxia, dyskinesia	Johannesen *et al*. (2018)
G550R (*unknown*)	Generalized epilepsy, autism spectrum disorder	Wang *et al*. (2016), Mattison *et al*. (2018)
R566H (*inherited*)	Generalized epilepsy, learning disorder, non-specific dysmorphisms	Posar & Visconti (2019)

*Pathogenic mutations in* SLC6A1 *listed with the associated clinical features and inheritance pattern*.

## Abbreviations

4-PBA: 4-phenylbutyric acid
ADHD: attention deficit hyperactivity disorder
BGT-1: betaine/GABA transporter 1
CRT-1: creatine transporter 1
DAT: dopamine transporter
DTDS: dopamine transporter deficiency syndrome
ER: endoplasmic reticulum
ERAD: ER associated degradation
GABA: γ-aminobutyric acid
GAT-1: GABA transporter 1
GLUT-1: glucose transporter 1
GLYT-2: glycine transporter 2
HSP: heat shock proteins
ID: intellectual disability
iPSC: induced pluripotent stem cells
KO: knockout
NET: norepinephrine transporter
SERT: serotonin transporter
SLC6: solute carrier 6
SNARE: soluble NSF attachment protein (SNAP) receptor
TM: transmembrane (domain)
VGAT: vesicular GABA transporter.

## References

[R1] Aguilar JI, Cheng MH, Font J, Schwartz AC, Ledwitch K, Duran A (2021). Psychomotor Impairments and Therapeutic Implications Revealed by a Mutation Associated with Infantile Parkinsonism-Dystonia. Elife.

[R2] Ahring PK, Liao VWY, Gardella E, Johannesen KM, Krey I, Selmer KK (2021). Gain-of-function Variants in GABRD Reveal a Novel Pathway for Neurodevelopmental Disorders and Epilepsy. Brain.

[R3] Asjad HMM, Kasture A, El-Kasaby A, Sackel M, Hummel T, Freissmuth M (2017). Pharmacochaperoning in a Drosophila Model System Rescues Human Dopamine Transporter Variants Associated with Infantile/juvenile Parkinsonism. J Biol Chem.

[R4] Avoli M (1995). Feline Generalized Penicillin Epilepsy. Ital. J Neuro Sci.

[R5] Awapara J, Landua AJ, Fuerst R, Seale B (1950). FREE γ-AMINOBUTYRIC ACID IN BRAIN. J Biol Chem.

[R6] Baraban SC, Taylor MR, Castro PA, Baier H (2005). Pentylenetetrazole Induced Changes in Zebrafish Behavior, Neural Activity and C-Fos Expression. Neuroscience.

[R7] Battini R, Chilosi AM, Casarano M, Moro F, Comparini A, Alessandrì MG (2011). Language Disorder with Mild Intellectual Disability in a Child Affected by a Novel Mutation of SLC6A8 Gene. Mol Genet Metab.

[R8] Beerepoot P, Lam VM, Salahpour A (2016). Pharmacological Chaperones of the Dopamine Transporter Rescue Dopamine Transporter Deficiency Syndrome Mutations in Heterologous Cells. J Biol Chem.

[R9] Bennett ER, Kanner BI (1997). The Membrane Topology of GAT-1, a (Na++ Cl-)-Coupled γ-Aminobutyric Acid Transporter from Rat Brain. J Biol Chem.

[R10] Bernstein EM, Quick MW (1999). Regulation of γ-Aminobutyric Acid (GABA) Transporters by Extracellular GABA. J Biol Chem.

[R11] Bhat S, El-Kasaby A, Freissmuth M, Sucic S (2021). Functional and Biochemical Consequences of Disease Variants in Neurotransmitter Transporters: A Special Emphasis on Folding and Trafficking Deficits. Pharmacol Ther.

[R12] Bhat S, Hasenhuetl PS, Kasture A, El-Kasaby A, Baumann MH, Blough BE (2017). Conformational State Interactions Provide Clues to the Pharmacochaperone Potential of Serotonin Transporter Partial Substrates. J Biol Chem.

[R13] Bowling KM, Thompson ML, Amaral MD, Finnila CR, Hiatt SM, Engel KL (2017). Genomic Diagnosis for Children with Intellectual Disability And/or Developmental Delay. Genome Med.

[R14] Bragina L, Marchionni I, Omrani A, Cozzi A, Pellegrini-Giampietro DE, Cherubini E (2008). GAT-1 Regulates Both Tonic and Phasic GABAAreceptor-Mediated Inhibition in the Cerebral Cortex. J Neurochem.

[R15] Brand AH, Perrimon N (1993). Targeted Gene Expression as a Means of Altering Cell Fates and Generating Dominant Phenotypes. Development.

[R16] Cai K, Wang J, Eissman J, Wang J, Nwosu G, Shen W (2019). A Missense Mutation in SLC6A1 Associated with Lennox-Gastaut Syndrome Impairs GABA Transporter 1 Protein Trafficking and Function. Exp Neurol.

[R17] Cai Y-Q, Cai G-Q, Liu G-X, Cai Q, Shi J-H, Shi J (2006). Mice with Genetically Altered GABA Transporter Subtype I (GAT1) Expression Show Altered Behavioral Responses to Ethanol. J Neurosci Res.

[R18] Carta E, Chung S-K, James VM, Robinson A, Gill JL, Remy N (2012). Mutations in the GlyT2 Gene (SLC6A5) Are a Second Major Cause of Startle Disease. J BiolChem.

[R19] Carvill GL, McMahon JM, Schneider A, Zemel M, Myers CT, Saykally J (2015). Mutations in the GABA Transporter SLC6A1 Cause Epilepsy with Myoclonic-Atonic Seizures. Am J Hum Genet.

[R20] Chaudhuri TK, Paul S (2006). Protein-misfolding Diseases and Chaperone-Based Therapeutic Approaches. FEBS J.

[R21] Chen N-H, Reith MEA, Quick MW (2004). Synaptic Uptake and beyond: The Sodium- and Chloride-dependent Neurotransmitter Transporter Family SLC6. Pflügers Archiv Eur. J Physiol.

[R22] Chiu C-S, Brickley S, Jensen K, Southwell A, Mckinney S, Cull-Candy S (2005). GABA Transporter Deficiency Causes Tremor, Ataxia, Nervousness, and Increased GABA-Induced Tonic Conductance in Cerebellum. J Neurosci.

[R23] Chiu C-S, Jensen K, Sokolova I, Wang D, Li M, Deshpande P (2002). Number, Density, and Surface/cytoplasmic Distribution of GABA Transporters at Presynaptic Structures of Knock-In Mice Carrying GABA Transporter Subtype 1-green Fluorescent Protein Fusions. J Neurosci.

[R24] Conti F, Zuccarello LV, Barbaresi P, Minelli A, Brecha NC, Melone M (1999). Neuronal, Glial, and Epithelial Localization of Gamma-Aminobutyric Acid Transporter 2, a High-Affinity Gamma-Aminobutyric Acid Plasma Membrane Transporter, in the Cerebral Cortex and Neighboring Structures. J Comp Neurol.

[R25] Cope DW, Di Giovanni G, Fyson SJ, Orbán G, Errington AC, Lőrincz ML (2009). Enhanced Tonic GABAA Inhibition in Typical Absence Epilepsy. Nat Med.

[R26] Deken SL, Beckman ML, Boos L, Quick MW (2000). Transport Rates of GABA Transporters: Regulation by the N-Terminal Domain and Syntaxin 1A. Nat Neurosci.

[R27] Devries S, Mulder M, Charron JG, Prokop JW, Mark PR (2020). SLC6A1 G443D Associated with Developmental Delay and Epilepsy. Cold Spring Harb Mol Case Stud.

[R28] El-Kasaby A, Kasture A, Koban F, Hotka M, Asjad HMM, Kubista H (2019). Rescue by 4-phenylbutyrate of Several Misfolded Creatine Transporter-1 Variants Linked to the Creatine Transporter Deficiency Syndrome. Neuropharmacology.

[R29] El-Kasaby A, Koban F, Sitte HH, Freissmuth M, Sucic S (2014). A Cytosolic Relay of Heat Shock Proteins HSP70-1A and HSP90β Monitors the Folding Trajectory of the Serotonin Transporter. J Biol Chem.

[R30] Engel JE, Wu C-F (1994). Altered Mechanoreceptor Response in Drosophila Bang-Sensitive Mutants. J Comp Physiol A.

[R31] Farr CV, El-Kasaby A, Freissmuth M, Sucic S (2020). The Creatine Transporter Unfolded: A Knotty Premise in the Cerebral Creatine Deficiency Syndrome. Front Synaptic Neurosci.

[R32] Freissmuth M, Stockner T, Sucic S (2017). SLC6 Transporter Folding Diseases and Pharmacochaperoning. Handb Exp Pharmacol.

[R33] Ganetzky B (2000). Genetic Analysis of Ion Channel Dysfunction in Drosophila. Kidney Int.

[R34] Ganetzky B, Wu C-F (1982). Indirect Suppression Involving Behavioral Mutants with Altered Nerve Excitability in Drosophila melanogaster. Genetics.

[R35] Germain DP, Hughes DA, Nicholls K, Bichet DG, Giugliani R, Wilcox WR (2016). Treatment of Fabry’s Disease with the Pharmacologic Chaperone Migalastat. N Engl J Med.

[R36] Ghosh SG, Becker K, Huang H, Dixon-Salazar T, Chai G, Salpietro V (2018). Biallelic Mutations in ADPRHL2, Encoding ADP-Ribosylhydrolase 3, Lead to a Degenerative Pediatric Stress-Induced Epileptic Ataxia Syndrome. Am J Hum Genet.

[R37] Goodspeed K, Pérez-Palma E, Iqbal S, Cooper D, Scimemi A, Johannesen KM (2020). Current Knowledge of SLC6A1-Related Neurodevelopmental Disorders. Brain Commun.

[R38] Gratz SJ, Rubinstein CD, Harrison MM, Wildonger J, O’Connor-Giles KM (2015). CRISPR-Cas9 Genome Editing in Drosophila. Curr Protoc Mol Biol.

[R39] Hahn MK, Robertson D, Blakely RD (2003). A Mutation in the Human Norepinephrine Transporter Gene (SLC6A2) Associated with Orthostatic Intolerance Disrupts Surface Expression of Mutant and Wild-type Transporters. J Neurosci.

[R40] Halvorsen M, Petrovski S, Shellhaas R, Tang Y, Crandall L, Goldstein D (2016). Mosaic Mutations in Early-Onset Genetic Diseases. Genet Med.

[R41] Höglund PJ, Adzic D, Scicluna SJ, Lindblom J, Fredriksson R (2005). The Repertoire of Solute Carriers of Family 6: Identification of New Human and Rodent Genes. Biochem Biophysical Res Commun.

[R42] Islam MP, Herman GE, de los Reyes EC (2018). Language Regression in an Atypical SLC6A1 Mutation. Semin Pediatr Neurol.

[R43] Jensen K, Chiu C-S, Sokolova I, Lester HA, Mody I (2003). GABA Transporter-1 (GAT1)-Deficient Mice: Differential Tonic Activation of GABAA versus GABAB Receptors in the hippocampus. J Neurophysiol.

[R44] Johannesen KM, Gardella E, Linnankivi T, Courage C, de Saint Martin A, Lehesjoki A-E (2018). Defining the Phenotypic Spectrum of SLC6A1mutations. Epilepsia.

[R45] Kahen A, Kavus H, Geltzeiler A, Kentros C, Taylor C, Brooks E (2021). Neurodevelopmental Phenotypes Associated with Pathogenic Variants in SLC6A1. J Med Genet.

[R46] Kang J-Q (2017). Defects at the Crossroads of GABAergic Signaling in Generalized Genetic Epilepsies. Epilepsy Res.

[R47] Kasture AS, Bartel D, Steinkellner T, Sucic S, Hummel T, Freissmuth M (2019). Distinct Contribution of Axonal and Somatodendritic Serotonin Transporters in Drosophila Olfaction. Neuropharmacol.

[R48] Kasture A, El-Kasaby A, Szöllősi D, Asjad HMM, Grimm A, Stockner T (2016). Functional Rescue of a Misfolded Drosophila melanogaster Dopamine Transporter Mutant Associated with a Sleepless Phenotype by Pharmacological Chaperones. J Biol Chem.

[R49] Kasture A, Hummel T, Sucic S, Freissmuth M (2018). Big Lessons from Tiny Flies: Drosophila melanogaster as a Model to Explore Dysfunction of Dopaminergic and Serotonergic Neurotransmitter Systems. Ijms.

[R50] Kasture A, Stockner T, Freissmuth M, Sucic S (2017). An Unfolding story: Small Molecules Remedy Misfolded Monoamine Transporters. Int J Biochem Cel Biol.

[R51] Kim D, Song I, Keum S, Lee T, Jeong M-J, Kim S-S (2001). Lack of the Burst Firing of Thalamocortical Relay Neurons and Resistance to Absence Seizures in Mice Lacking α1G T-type Ca2+ Channels. Neuron.

[R52] Koban F, El-Kasaby A, Häusler C, Stockner T, Simbrunner BM, Sitte HH (2015). A Salt Bridge Linking the First Intracellular Loop With the C Terminus Facilitates the Folding of the Serotonin Transporter. J Biol Chem.

[R53] Kristensen AS, Andersen J, Jørgensen TN, Sørensen L, Eriksen J, Loland CJ (2011). SLC6 Neurotransmitter Transporters: Structure, Function, and Regulation. Pharmacol Rev.

[R54] Kuebler D, Tanouye MA (2000). Modifications of Seizure Susceptibility inDrosophila. J Neurophysiol.

[R55] Kurian MA, Zhen J, Cheng S-Y, Li Y, Mordekar SR, Jardine P (2009). Homozygous Loss-Of-Function Mutations in the Gene Encoding the Dopamine Transporter Are Associated with Infantile Parkinsonism-Dystonia. J Clin Invest.

[R56] Lamb AM, Walker EA, Wittkopp PJ (2017). Tools and Strategies for Scarless Allele Replacement in Drosophila Using CRISPR/Cas9. Fly.

[R57] Landrum MJ, Lee JM, Benson M, Brown GR, Chao C, Chitipiralla S (2018). ClinVar: Improving Access to Variant Interpretations and Supporting Evidence. Nucleic Acids Res.

[R58] LaRoche SM, Helmers SL (2004). The New Antiepileptic Drugs. Jama.

[R59] Larsen J, Johannesen KM, Ek J, Tang S, Marini C, Blichfeldt S (2015). The Role ofSLC2A1mutations in Myoclonic Astatic Epilepsy and Absence Epilepsy, and the Estimated Frequency of GLUT1 Deficiency Syndrome. Epilepsia.

[R60] Lee J, Wu C-F (2002). Electroconvulsive Seizure Behavior inDrosophila: Analysis of the Physiological Repertoire Underlying a Stereotyped Action Pattern in Bang-Sensitive Mutants. J Neurosci.

[R61] Liu G-X, Cai G-Q, Cai Y-Q, Sheng Z-J, Jiang J, Mei Z (2007a). Reduced Anxiety and Depression-like Behaviors in Mice Lacking GABA Transporter Subtype 1. Neuropsychopharmacol.

[R62] Liu G-X, Liu S, Cai G-Q, Sheng Z-J, Cai Y-Q, Jiang J (2007b). Reduced Aggression in Mice Lacking GABA Transporter Subtype 1. J Neurosci Res.

[R63] Liu Z, Zhang N, Zhang Y, Du Y, Zhang T, Li Z (2018). Prioritized High-Confidence Risk Genes for Intellectual Disability Reveal Molecular Convergence during Brain Development. Front Genet.

[R64] Loo TW, Clarke DM (2007). Chemical and Pharmacological Chaperones as New Therapeutic Agents. Expert Rev Mol Med.

[R65] Lucariello M, Vidal E, Vidal S, Saez M, Roa L, Huertas D (2016). Whole Exome Sequencing of Rett Syndrome-like Patients Reveals the Mutational Diversity of the Clinical Phenotype. Hum Genet.

[R66] Macpherson LJ, Zaharieva EE, Kearney PJ, Alpert MH, Lin T-Y, Turan Z (2015). Dynamic Labelling of Neural Connections in Multiple Colours by Trans-synaptic Fluorescence Complementation. Nat Commun.

[R67] Manivannan SN, Roovers J, Smal N, Myers CT, Turkdogan D, Roelens F (2021). De Novo FZR1 Loss-Of-Function Variants Cause Developmental and Epileptic Encephalopathies. Brain.

[R68] Marvin JS, Shimoda Y, Magloire V, Leite M, Kawashima T, Jensen TP (2019). A Genetically Encoded Fluorescent Sensor for In Vivo Imaging of GABA. Nat Methods.

[R69] Mattison KA, Butler KM, Inglis GAS, Dayan O, Boussidan H, Bhambhani V (2018). SLC6A1 Variants Identified in Epilepsy Patients Reduce γ-aminobutyric Acid Transport. Epilepsia.

[R70] Mazaud D, Kottler B, Gonçalves-Pimentel C, Proelss S, Tüchler N, Deneubourg C (2019). Transcriptional Regulation of the glutamate/GABA/glutamine Cycle in Adult Glia Controls Motor Activity and Seizures in drosophila. J Neurosci.

[R71] Mermer F, Poliquin S, Rigsby K, Rastogi A, Shen W, Romero-Morales A (2021). Common Molecular Mechanisms of SLC6A1 Variant-Mediated Neurodevelopmental Disorders in Astrocytes and Neurons. Brain.

[R72] Minelli A, Brecha N, Karschin C, DeBiasi S, Conti F (1995). GAT-1, a High-Affinity GABA Plasma Membrane Transporter, Is Localized to Neurons and Astroglia in the Cerebral Cortex. J Neurosci.

[R73] Minelli A, DeBiasi S, Brecha NC, Vitellaro Zuccarello L, Conti F (1996). GAT-3, a High-Affinity GABA Plasma Membrane Transporter, Is Localized to Astrocytic Processes, and it Is Not Confined to the Vicinity of GABAergic Synapses in the Cerebral Cortex. J Neurosci.

[R74] Montgomery TS, Steinkellner T, Sucic S, Koban F, Schüchner S, Ogris E (2014). Axonal Targeting of the Serotonin Transporter in Cultured Rat Dorsal Raphe Neurons Is Specified by SEC24C-Dependent Export from the Endoplasmic Reticulum. J Neurosci.

[R75] Moss FJ, Imoukhuede PI, Scott K, Hu J, Jankowsky JL, Quick MW (2009). GABA Transporter Function, Oligomerization State, and Anchoring: Correlates with Subcellularly Resolved FRET. J Gen Physiol.

[R76] Muthukumar AK, Stork T, Freeman MR (2014). Activity-dependent Regulation of Astrocyte GAT Levels during Synaptogenesis. Nat Neurosci.

[R77] Ng J, Barral S, de la Fuente Barrigon C, Lignani G, Erdem FA, Wallings R (2021). Gene Therapy Restores Dopamine Transporter Expression and Ameliorates Pathology in iPSC and Mouse Models of Infantile Parkinsonism. Sci Transl Med.

[R78] Ng J, Heales SJR, Kurian MA (2014). Clinical Features and Pharmacotherapy of Childhood Monoamine Neurotransmitter Disorders. Pediatr Drugs.

[R79] Noebels JL (2003). The Biology of Epilepsy Genes. Annu Rev Neurosci.

[R80] Palmer S, Towne MC, Pearl PL, Pelletier RC, Genetti CA, Shi J (2016). SLC6A1 Mutation and Ketogenic Diet in Epilepsy with Myoclonic-Atonic Seizures. Pediatr Neurol.

[R81] Pavlidis P, Tanouye M (1995). Seizures and Failures in the Giant Fiber Pathway of Drosophila Bang-Sensitive Paralytic Mutants. J Neurosci.

[R82] Perlmutter DH (2002). Chemical Chaperones: a Pharmacological Strategy for Disorders of Protein Folding and Trafficking. Pediatr Res.

[R83] Poliquin S, Hughes I, Shen W, Mermer F, Wang J, Mack T (2021). Genetic Mosaicism, Intrafamilial Phenotypic Heterogeneity, and Molecular Defects of a Novel Missense SLC6A1 Mutation Associated with Epilepsy and ADHD. Exp Neurol.

[R84] Posar A, Visconti P (2019). Mild Phenotype Associated with SLC6A1 Gene Mutation: A Case Report with Literature Review. J Pediatr Neurosci.

[R85] Quick MW, Hu J, Wang D, Zhang H-Y (2004). Regulation of a γ-Aminobutyric Acid Transporter by Reciprocal Tyrosine and Serine Phosphorylation. J Biol Chem.

[R86] Roberts E, Frankel S (1950). γ-AMINOBUTYRIC ACID IN BRAIN: ITS FORMATION FROM GLUTAMIC ACID. J Biol Chem.

[R87] Rauch A, Wieczorek D, Graf E, Wieland T, Endele S, Schwarzmayr T (2012). Range of Genetic Mutations Associated with Severe Non-syndromic Sporadic Intellectual Disability: An Exome Sequencing Study. The Lancet.

[R88] Rees E, Han J, Han J, Morgan J, Carrera N, Escott-Price V (2020). De Novo mutations Identified by Exome Sequencing Implicate Rare Missense Variants in SLC6A1 in Schizophrenia. Nat Neurosci.

[R89] Rees MI, Harvey K, Pearce BR, Chung S-K, Duguid IC, Thomas P (2006). Mutations in the Gene Encoding GlyT2 (SLC6A5) Define a Presynaptic Component of Human Startle Disease. Nat Genet.

[R90] Reiter LT, Potocki L, Chien S, Gribskov M, Bier E (2001). A Systematic Analysis of Human Disease-Associated Gene Sequences in Drosophila melanogaster. Genome Res.

[R91] Roth FC, Draguhn A (2012). GABA Metabolism and Transport: Effects on Synaptic Efficacy. Neural Plasticity.

[R92] Rowley NM, Madsen KK, Schousboe A, Steve White H (2012). Glutamate and GABA Synthesis, Release, Transport and Metabolism as Targets for Seizure Control. Neurochem Int.

[R93] Rubin GM, Spradling AC (1982). Genetic Transformation of Drosophila with Transposable Element Vectors. Science.

[R94] Ryoo HD, Li J, Kang M-J (2013). Drosophila XBP1 Expression Reporter Marks Cells under Endoplasmic Reticulum Stress and with High Protein Secretory Load. PLoS One.

[R95] Salomons GS, Van Dooren SJM, Verhoeven NM, Cecil KM, Ball WS, Degrauw TJ (2001). X-linked Creatine-Transporter Gene (SLC6A8) Defect: A New Creatine-Deficiency Syndrome. Am J Hum Genet.

[R96] Sanders SJ, Murtha MT, Gupta AR, Murdoch JD, Raubeson MJ, Willsey AJ (2012). De Novo mutations Revealed by Whole-Exome Sequencing Are Strongly Associated with Autism. Nature.

[R97] Satterstrom FK, Kosmicki JA, Wang J, Breen MS, De Rubeis S, An JY (2020). Large-Scale Exome Sequencing Study Implicates Both Developmental and Functional Changes in the Neurobiology of Autism. Cell.

[R98] Schmid JA, Scholze P, Kudlacek O, Freissmuth M, Singer EA, Sitte HH (2001). Oligomerization of the Human Serotonin Transporter and of the Rat GABA Transporter 1 Visualized by Fluorescence Resonance Energy Transfer Microscopy in Living Cells. J Biol Chem.

[R99] Scholze P, Freissmuth M, Sitte HH (2002). Mutations within an Intramembrane Leucine Heptad Repeat Disrupt Oligomer Formation of the Rat GABA Transporter 1. J Biol Chem.

[R100] Schousboe A, Madsen KK, Barker-Haliski ML, White HS (2014). The GABA Synapse as a Target for Antiepileptic Drugs: A Historical Overview Focused on GABA Transporters. Neurochem Res.

[R101] Scimemi A (2014). Structure, Function, and Plasticity of GABA Transporters. Front Cel Neurosci.

[R102] Shannon JR, Flattem NL, Jordan J, Jacob G, Black BK, Biaggioni I (2000). Orthostatic Intolerance and Tachycardia Associated with Norepinephrine-Transporter Deficiency. N Engl J Med.

[R103] Shearin HK, Quinn CD, Mackin RD, Macdonald IS, Stowers RS (2018). t-GRASP, a Targeted GRASP for Assessing Neuronal Connectivity. J Neurosci Methods.

[R104] Song J, Tanouye M (2008). From Bench to Drug: Human Seizure Modeling Using Drosophila. Prog Neurobiol.

[R105] Soragna A, Bossi E, Giovannardi S, Pisani R, Peres A (2005). Functionally Independent Subunits in the Oligomeric Structure of the GABA Cotransporter rGAT1. Cell Mol Life Sci.

[R106] Soustelle L, Besson M-T, Rival T, Birman S (2002). Terminal Glial Differentiation Involves Regulated Expression of the Excitatory Amino Acid Transporters in the Drosophila Embryonic CNS. Dev Biol.

[R107] Stilwell GE, Saraswati S, Littleton JT, Chouinard SW (2006). Development of aDrosophilaseizure Model Forin Vivohigh-Throughput Drug Screening. Eur J Neurosci.

[R108] Stork T, Sheehan A, Tasdemir-Yilmaz OE, Freeman MR (2014). Neuron-Glia Interactions through the Heartless Fgf Receptor Signaling Pathway Mediate Morphogenesis of drosophila Astrocytes. Neuron.

[R109] Sucic S, Kasture A, Mazhar Asjad HM, Kern C, El-Kasaby A, Freissmuth M (2016). When Transporters Fail to Be Transported:how to rescue Folding-Deficient SLC6 Transporters. J Neurol Neuromedicine.

[R110] Tang S, Addis L, Smith A, Topp SD, Pendziwiat M, Mei D (2020). Phenotypic and Genetic Spectrum of Epilepsy with Myoclonic Atonic Seizures. Epilepsia.

[R111] Thimgan MS, Berg JS, Stuart AE (2006). Comparative Sequence Analysis and Tissue Localization of Members of the SLC6 Family of Transporters in adultDrosophila Melanogaster. J Exp Biol.

[R112] van de Kamp JM, Mancini GM, Salomons GS (2014). X-linked Creatine Transporter Deficiency: Clinical Aspects and Pathophysiology. J Inherit Metab Dis.

[R113] Waagepetersen HS, Sonnewald U, Schousboe A (1999). The GABA Paradox. J Neurochem.

[R114] Wainwright CE, Elborn JS, Ramsey BW, Marigowda G, Huang X, Cipolli M (2015). Lumacaftor-Ivacaftor in Patients with Cystic Fibrosis Homozygous for Phe508del CFTR. N Engl J Med.

[R115] Wang J, Poliquin S, Mermer F, Eissman J, Delpire E, Wang J (2020). Endoplasmic Reticulum Retention and Degradation of a Mutation in SLC6A1 Associated with Epilepsy and Autism. Mol Brain.

[R116] Wang T, Guo H, Xiong B, Stessman HAF, Wu H, Coe BP (2016). De Novo genic Mutations Among a Chinese Autism Spectrum Disorder Cohort. Nat Commun.

[R117] Williams SN, Locke CJ, Braden AL, Caldwell KA, Caldwell GA (2004). Epileptic-like Convulsions Associated with LIS-1 in the Cytoskeletal Control of Neurotransmitter Signaling in Caenorhabditis elegans. Hum Mol Genet.

[R118] Wu Y, Wang W, Díez-Sampedro A, Richerson GB (2007). Nonvesicular Inhibitory Neurotransmission via Reversal of the GABA Transporter GAT-1. Neuron.

[R119] Xu YF, Cai YQ, Cai GQ, Jiang J, Sheng ZJ, Wang ZG (2008). Hypoalgesia in Mice Lacking GABA Transporter Subtype 1. J Neurosci Res.

[R120] Yan X-X, Cariaga WA, Ribak CE (1997). Immunoreactivity for GABA PlasmaMembrane Transporter, GAT-1, in the Developing Rat Cerebral Cortex: Transient Presence in the Somata of Neocortical and Hippocampal Neurons. Dev Brain Res.

[R121] Yap ZY, Efthymiou S, Seiffert S, Vargas Parra K, Lee S, Nasca A (2021). Bi-allelic Variants in OGDHL Cause a Neurodevelopmental Spectrum Disease Featuring Epilepsy, Hearing Loss, Visual Impairment, and Ataxia. Am J Hum Genet.

[R122] Yuen RK, Merico D, Cao H, Pellecchia G, Alipanahi B, Thiruvahindrapuram B (2016). Genome-wide Characteristics of De Novo Mutations in Autism. Npj Genomic Med.

[R123] Zhou Y, Danbolt NC (2013). GABA and Glutamate Transporters in Brain. Front Endocrinol.

[R124] Zhou Y, Holmseth S, Hua R, Lehre AC, Olofsson AM, Poblete-Naredo I (2012). The Betaine-GABA Transporter (BGT1, Slc6a12) Is Predominantly Expressed in the Liver and at Lower Levels in the Kidneys and at the Brain Surface. Am J Physiology-Renal Physiol.

[R125] Zhu X-M, Ong W-Y (2004). A Light and Electron Microscopic Study of Betaine/GABA Transporter Distribution in the Monkey Cerebral Neocortex and hippocampus. J Neurocytol.

